# Pyrotinib and trastuzumab plus palbociclib and fulvestrant in HR+/HER2+ breast cancer patients with brain metastasis

**DOI:** 10.1038/s41523-024-00646-2

**Published:** 2024-06-13

**Authors:** Dongshao Chen, Fei Xu, Yongkui Lu, Wen Xia, Caiwen Du, Dun Xiong, Dong Song, Yanxia Shi, Zhongyu Yuan, Qiufan Zheng, Kuikui Jiang, Xin An, Cong Xue, Jiajia Huang, Xiwen Bi, Meiting Chen, Jingmin Zhang, Shusen Wang, Ruoxi Hong

**Affiliations:** 1 Department of Medical Oncology, State Key Laboratory of Oncology in South China, Guangdong Provincial Clinical Research Center for Cancer, Sun Yat-sen University Cancer Center, Guangzhou, 510060 P.R. China; 2 Department of Breast, Bone & Soft Tissue Oncology, Guangxi Medical University Cancer Hospital, Nanning, Guangxi China; 3 National Cancer Center/National Clinical Research Center for Cancer/Cancer Hospital & Shenzhen Hospital, Chinese Academy of Medical Sciences and Peking Union Medical College, Shenzhen, P.R. China; 4 Department of Oncology, Puer People’s Hospital, Puer, Yunnan China; 5 Department of Breast Surgery, The First Hospital of Jilin University, Changchun, China

**Keywords:** Breast cancer, Targeted therapies

## Abstract

Human epidermal growth factor receptor 2-positive (HER2+) breast cancer (BC) patients are at a high risk of developing metastases in the brain. However, research focusing on treatment strategies for hormonal receptor positive (HR+), HER2+ BC patients with brain metastases (BM) remains limited. Thus, a multi-center, prospective trial was conducted in China. Women over the age of 18 who were naive to whole brain radiotherapy and had estrogen receptor (ER)/progesterone-receptor (PgR) positive, HER2+ BM were treated with palbociclib, fulvestrant, trastuzumab and pyrotinib, until disease progression or the development of intolerable side effects. The primary endpoint was objective response rate (ORR) in the central nervous system (CNS). This ongoing study is still recruiting participants and is registered with ClinicalTrials.gov (NCT04334330). This report presents the findings from an interim analysis. From December 4, 2020, to November 2, 2022, 15 patients were enrolled. Among the 14 patients who were evaluable for clinical response, the ORR was 35.7% (95% CI: 12.8–64.9%), with a CNS–ORR of 28.6% (95% CI: 8.4–58.1%). The median follow-up period was 6.3 months (range, 2.1–14.3 months), during which the median progression-free survival (PFS) was 10.6 months (95% CI: 4.3–16.9 months), and the median time to CNS progression was 8.5 months (95% CI: 5.9–11.1 months). The most common adverse event was diarrhea (93%), with 33% having grade 3 and 6.7% having grade 4. The study suggests that the combination of palbociclib, trastuzumab, pyrotinib and fulvestrant offers a promising chemo-free treatment strategy for HR+, HER2+ BC patients with BM.

## Introduction

Breast cancer (BC) ranks as the second most common cause of brain metastases (BM). It has been observed that BM occurs more frequently in HER2-positive (HER2+) metastatic breast cancer (MBC) (30–55% of patients) compared to HER2− BC^1,2^, and up to 50% of patients with HER2+ MBC succumb to central nervous system (CNS) progression^3^. Hence, BM presents a critical life-threatening challenge in patients with HER2+ MBC.

Systemic therapy, particularly when effective, holds promise in managing both intracranial and extracranial metastatic lesions. Targeting HER2 is a principal therapeutic strategy for HER2+ BC. Yet, managing BM in HER2+ BC with anti-HER2 monotherapy has not yielded satisfactory results. The median progression-free survival (PFS) of trastuzumab emtansine (T-DM1) in such cases ranged from 5^4^ to 6.1 months^5^. The CLEOPATRA^6^ and PHILA^7^ studies underscore that dual HER2 inhibition outperforms singular HER2 blockade in HER2+ MBC. Despite this, existing anti-HER2 treatments have limited efficacy in BM management, with CNS objective response rates (ORR) hovering around 10%^8,9^, highlighting the imperative for more integrative treatment modalities. The HER2CLIMB trial has demonstrated that supplementing trastuzumab and capecitabine with tucatinib can extend median CNS–PFS to 9.9 months^10^, although adverse events (AEs) were responsible for death in 1.5% of participants^11^. Thus, a low-toxic, efficient, and chemo-free treatment strategy is urgently needed.

Studies have shown that the absence of hormone receptor (HR) expression correlates with a higher risk of brain metastasis^12^. A recent pooled analysis indicated that compared to patients with HR−/HER2+ MBC, those with HR+/HER2+ were less likely to develop BM with an incidence rate ratio of 0.71 (95% CI: 0.64–0.78)^13^. Notably, approximately 50% of HER2+ group also express estrogen receptor (ER) and/or progesterone receptor (PR) and may benefit from endocrine therapy alongside anti-HER2 therapy^14^. Meanwhile, activation of cyclin D1-CDK4/6-RB transcriptional axis has been implicated in endocrine treatment resistance^14^. Research has highlighted that the integration of endocrine therapy with CDK4/6 inhibitors is an efficacious approach for treating patients with HR+ MBC^15,16^. CDK4/6 inhibitors, proven to overcome the therapeutic resistance of HER2 target therapy^17^, are presently under investigation for their applicability in the treatment of HER2+ BC. Preliminary results from the NA-PHER2 study show a clinical ORR of 97% before surgery and a pathological complete response rate of 27% at surgery with palbociclib in combination with trastuzumab, pertuzumab, and fulvestrant in preoperative ER+/HER2+ BC patients^18^. Moreover, ongoing clinical studies, such as the SOLTI-1303 PATRICIA and NCT03913234 studies, are evaluating the combination of HER2 targeted therapy with CDK4/6 inhibitors for advanced HER2+ BC. However, research focusing on treatment strategies for ER+/HER2+ MBC with BM remains limited.

Pyrotinib, an oral tyrosine kinase inhibitor (TKI) targeting HER2, was validated for its efficacy in the PHOEBE trial^19^ and subsequently received approval for HER2+ BC treatment in China in 2018^20^. When combined with the HER2-antibody trastuzumab, pyrotinib delivers a comprehensive inhibition of HER signaling. Palbociclib, a pioneering oral CDK4/6 inhibitor, has gained approval for treating HR+ BC in China. Notably, the combination of palbociclib and fulvestrant has demonstrated a statistically significant enhancement in overall survival (OS) for patients with HR+ BC in the PALOMA-3 study^21^.

This study represents the prospective investigation into the intracranial and extracranial therapeutic effects and safety of palbociclib, trastuzumab, pyrotinib, and fulvestrant in HR+/HER2+ MBC patients with BM. It aims to provide a chemo-free therapeutic strategy for clinical application, which might decrease the adverse effects of chemotherapy and increase the compliance of patients as well. Though still in progress, the interim analysis suggests promising efficacy of the study regimen for BCBM. Hence, we are reporting these preliminary findings, with the final results anticipated upon study completion.

## Results

### Patient characteristics

**Fig. 1:**
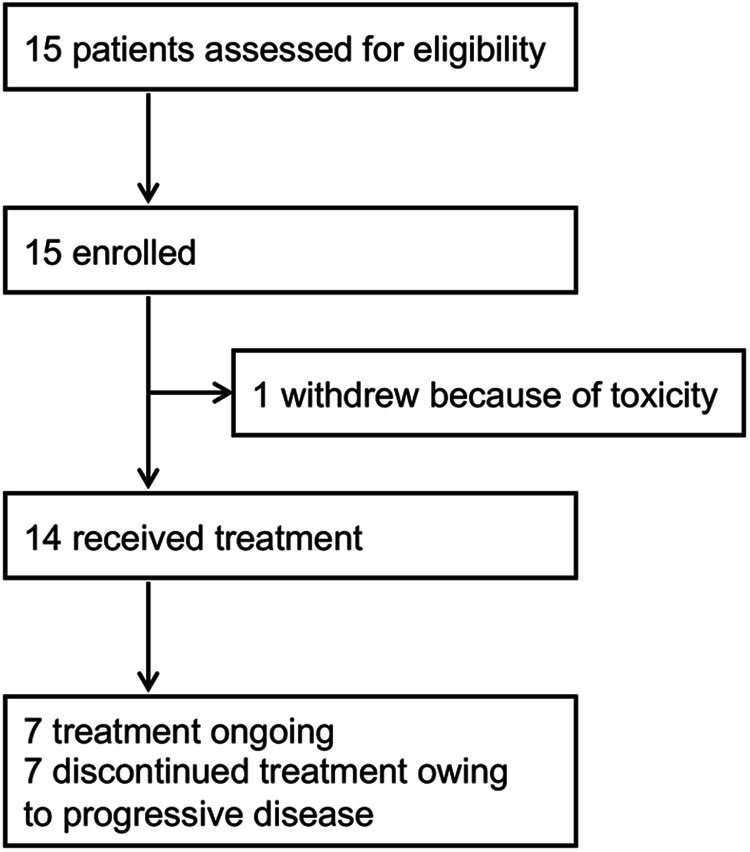
Trial profile.

From December 4, 2020, to November 2, 2022, we enrolled 15 patients across two of the three participating centres. As of Feb 3, 2023, seven patients (47%) remained on the study treatment. An equal proportion (47%) discontinued treatment due to disease progression, and one patient (6%) withdrew owing to toxicity without any post-baseline assessment; this patient, unfortunately, passed away three months after initiating treatment due to the swift advancement of her condition. 14 patients (93%) completed at least two treatment cycles (Fig. 1). Baseline characteristics of patients are listed in Table 1. The median follow-up duration was 6.3 months (range 2.14–14.29). A majority of the patients (87%) presented with both BM and extracranial lesions. In 73.3% of patients, the number of CNS lesions was between 2 and 5. In total, 86% of patients were identified as having BM during standard follow-up visits, with the remaining cases detected through symptoms indicative of CNS involvement, such as headaches and dizziness. The most frequent sites of extracranial disease included the lungs, liver, and bones. Notably, 11 patients had previously been treated with trastuzumab, including as part of adjuvant chemotherapy.

**Table 1:** Baseline characteristics

Characteristic		
Age, years, *n* (%)		
Median, range	55 (37–67)	
<60	12	(80)
≥60	3	(20)
Female sex	15	
*ECOG status, n (%)*		
0	0	(0)
1	13	(86.7)
2	2	(13.3)
Median follow-up time (months)	6.3 (2.14–14.29)	
*Sites of disease outside the CNS (not mutually exclusive)-no.*		
Lung	11	
Liver	4	
Bone	6	
Breast or chest wall	2	
Lymph nodes	12	
None	2	
*Number of CNS lesions, n (%)*		
1	1	(6.7)
2–5	11	(73.3)
>5	3	(20.0)
*BM identified at baseline, n (%)*		
Progression during prior treatment	8	(53.3)
Routine examination	5	(33.3)
Neurological symptoms	2	(13.3)
Previous therapies^*^-no.		
Trastuzumab	11	
Pertuzumab	6	
Lapatinib	2	
Other HER2 agents	2	
*Chemotherapy lines in the metastatic setting*^*†*^*-no. (%)*		
0	7	(46.7)
1	3	(20.0)
2	3	(20.0)
≥3	2	(13.3)

^†^Not including hormonal therapy.

^*^Including adjuvant therapy.

### Efficacy and survival

**Fig. 2:**
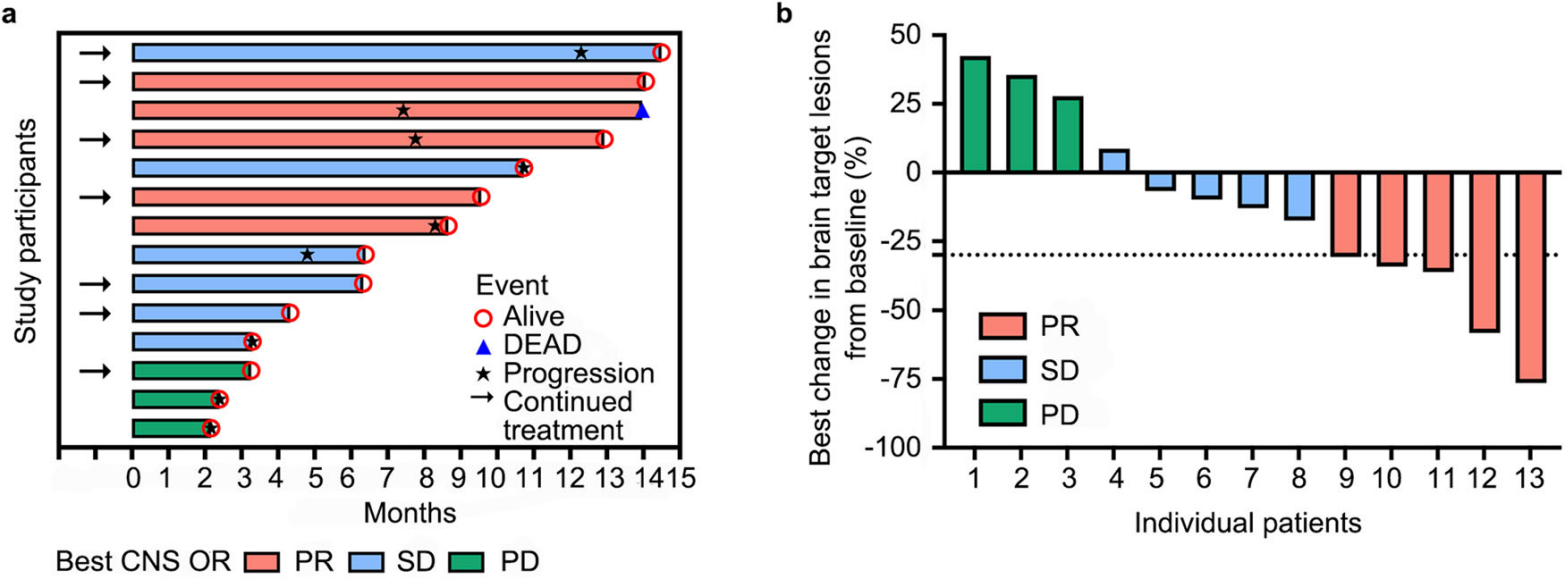
The best response and tumor change in CNS during the follow-ups. **a** Swimmer plot of the optimum response (PR/SD/PD) of each patient during the corresponding follow-up time (month). **b** Waterfall plot of tumor changes in CNS target lesion size. One patient had a stable disease, but the accurate tumor change was not evaluable due to the imaging examinations being conducted at a local hospital. PR partial response, PD progressive disease, SD stable disease.

**Fig. 3:**
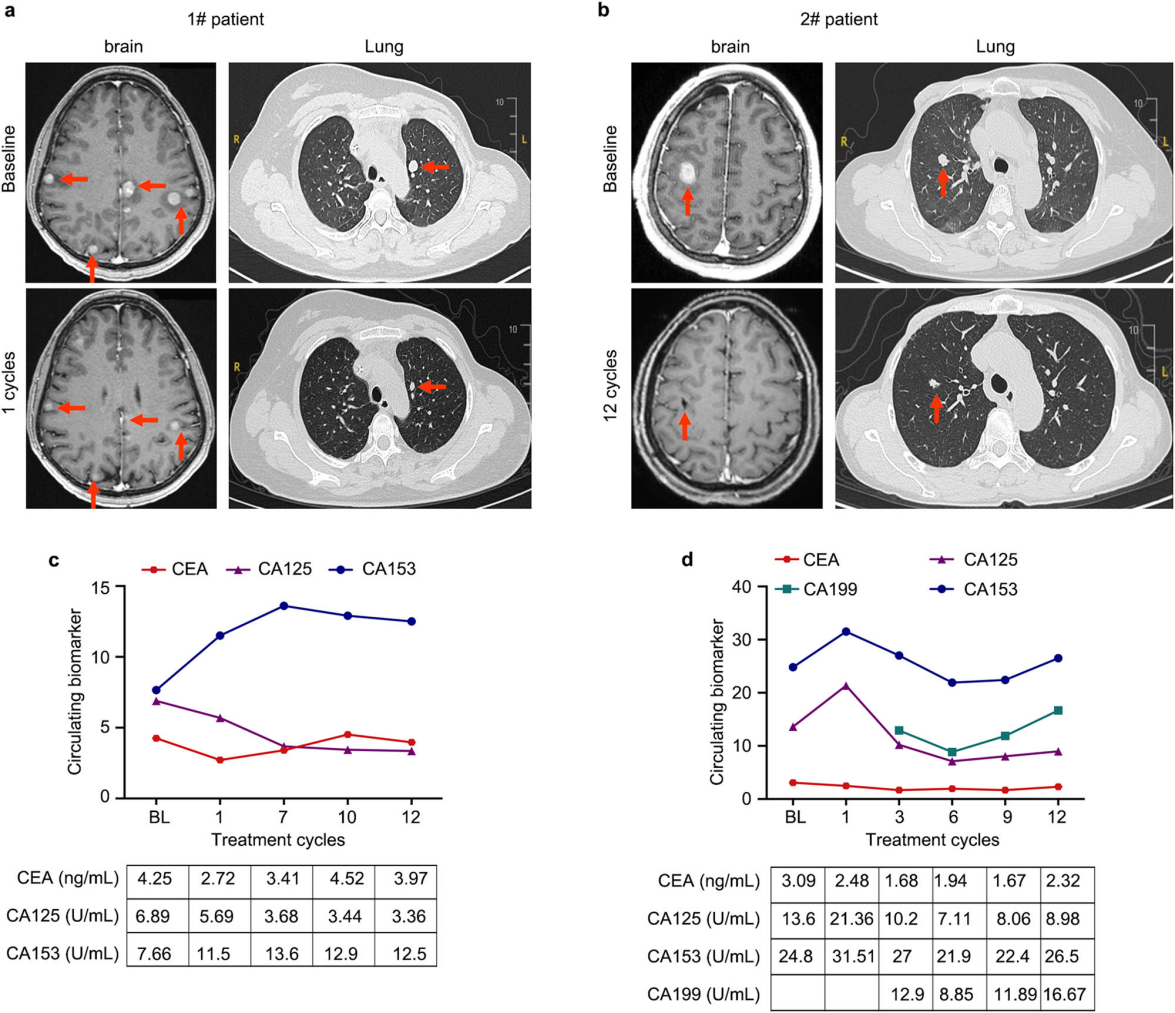
The clinical responses of two patients treated with palbociclib, trastuzumab, and pyrotinib with fulvestrant. **a** MRI/CT scans of tumor metastases in brain and lung during one cycles of combination treatment in patient 1. **b** MRI/CT scans of tumor metastases in brain and lung during 12 cycles of combination treatment in patient 2. **c**, **d** The dynamic change of CEA, CA125, CA153, and CA199 during combination treatment in patients 1 and 2.

Among the 14 patients assessed for clinical response, five (35.7%, 95%CI: 8.4–58.1%) experienced a CNS objective response, all of which were partial responses (PR). Six patients (42.9%) achieved stable disease (SD) as their best response, while three patients (21.4%) developed CNS progression at the first assessment (Table 2). The best response and the tumor changes in CNS target lesions during follow-up are shown in Fig. 2a, b as Swimmer plots and Waterfall, respectively. The efficacy of this scheme can be exemplified in Fig. 3, where after one treatment cycle for patient 1 and twelve cycles for patient 2, multiple targets or non-target lesions of intracranial and extracranial significantly reduced in size accompanied by decreased tumor biomarkers (Fig. 3a–d). Focusing on overall response rates, five patients (35.7%, 95% CI: 12.8–64.9%) showed PR, seven maintained SD, and two had progressive disease (PD) according to RECIST 1.1 criteria (Table 2). Among the seven patients who reached PD, the initial progression site was exclusively CNS in three (43%) patients and involved both CNS and extra-CNS lesions in four (57%) patients. Notably, no patients discontinued study treatment solely due to CNS progression without concurrent extracranial progression. For the five patients responding to treatment, the median duration from study inclusion to the first documented response was 2.0 months (range 1.3–3.0).

**Table 2:** The best objective response in assessable patients

	CNS objective response *n* (%)		Overall objective response *n* (%)	
ORR	5	(35.7)	5	(35.7)
CR	0		0	
PR	5	(35.7)	5	(35.7)
SD	6	(42.9)	7	(50.0)
PD	3	(21.4)	2	(14.3)
Total	14		14

**Fig. 4:**
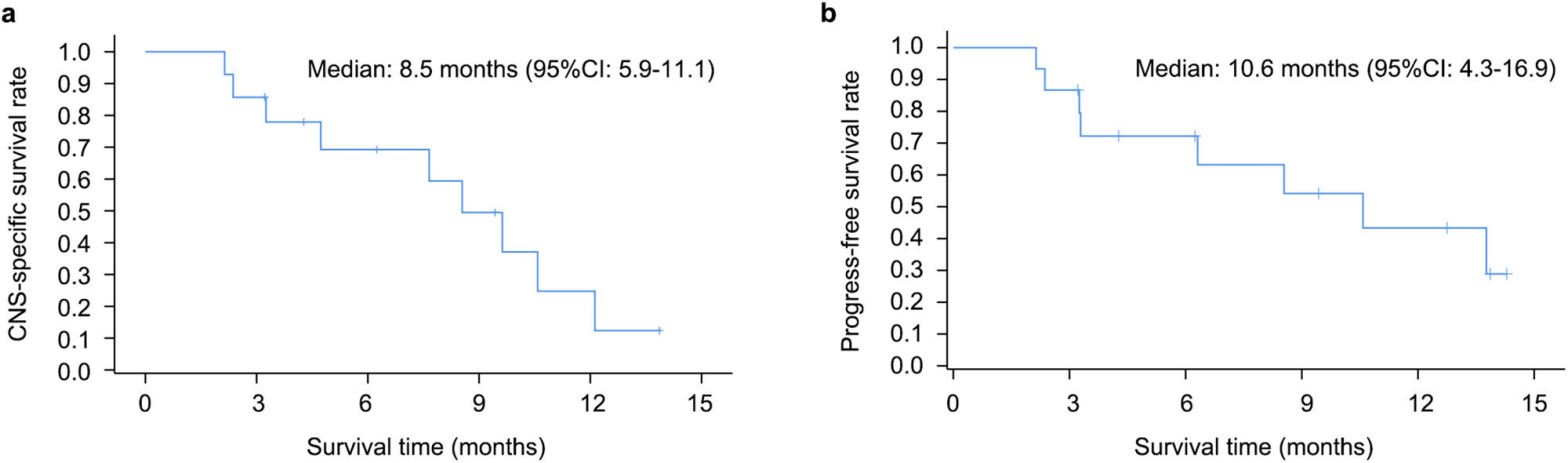
Kaplan-Meier estimates of progression-free survival. Time to CNS progression (**a**) and overall progression-free survival (**b**).

At a median follow-up of 6.3 months, the median time to CNS progression was 8.5 months (95% CI: 5.9–11.1 months; Fig. 4a), and the median PFS was 10.6 months (95% CI: 4.3–16.9 months; Fig. 4b). The 12-month overall survival rate stood at 43.3% (95% CI: 15.3–68.9%) and the clinical benefit rate (CBR) for nine patients with a follow-up period exceeding six months was remarkably high at 88.9% (95% CI: 51.8–99.7%).

### Treatment duration and safety

As of Feb 3, 2023, median number of treatment cycles was 8.3 (range 2.3–15.5). Nine patients (64%) received treatment for more than 6 months, and four patients (29%) underwent treatment for over a year. Treatment-emergent adverse events are listed in Table 3. The most common adverse event was diarrhea, with 53% experiencing grade 1–2, 33% facing grade 3, and 6.7% encountering grade 4 diarrhea. Grade 1–2 (53%) leukopenia, vomiting (47%), and anemia (40%) were also common, with the remainder of the toxicities occurring less frequently. The most common grade 3 treatment-emergent adverse event was diarrhea, with one (6.7%) leukopenia and one (6.7%) marrow suppression. The sole grade 4 adverse event reported was severe diarrhea, necessitating a dose reduction of pyrotinib to 320 mg in the second cycle. No treatment-related deaths occurred.

**Table 3:** Treatment-emergent adverse events

	Grade 1–2	Grade 3	Grade 4
Diarrhea	8 (53%)	5 (33%)	1 (6.7%)
leukopenia	8 (53%)	1 (6.7%)	0
Vomiting	7 (47%)	0	0
Anemia	6 (40%)	0	0
Nausea	4 (27%)	0	0
Fatigue	3 (20%)	0	0
Rash	3 (20%)	0	0
Neutropenia	3 (20%)	0	0
Anorexia	3 (20%)	0	0
Oral ulcer	2 (13%)	0	0
Dizziness	2 (13%)	0	0
Marrow suppression	2 (13%)	1 (6.7%)	0
Abdominal distension	1 (6.7%)	0	0
Headache	1 (6.7%)	0	0

Data are *n* (%). Grade 1–2 events occur in all patients and all grade 3 and 4 events are reported. Each patient was counted once for the highest grade of each event experienced.

Regarding dose modifications, 10 (67%) required a palbociclib dose reduction to 100 mg, and one (6.7%) had an additional reduction of palbociclib to 75 mg. Six (40%) patients required pyrotinib dose reductions, with three to 320 mg, one to 240 mg, and two initially to 320 mg followed by a further reduction to 240 mg. Diarrhea was the main cause leading to the dose adjustments of pyrotinib. Five (33%) patients experienced dose reductions of both palbociclib and pyrotinib. One (6.7%) patient discontinued the pyrotinib treatment because of grade 2 diarrhea and vomiting, and one (6.7%) ceased both palbociclib and pyrotinib following COVID-19 infection and associated grade 1–2 adverse events.

## Discussion

Our study aims to assess the combination of dual blockage of CDK4/6 and HER2 with endocrine therapy in patients with BM from HR+/HER2+ BC. It demonstrates that the combination of palbociclib, trastuzumab and pyrotinib with fulvestrant yields a CNS ORR of 35.7% (95% CI: 8.4–58.1%) and a median time to CNS progression of 8.5 months (95% CI: 5.9–11.1 months), suggesting the promising combination strategy for the treatment of BM in patients with HR+/HER2+ MBC.

BM occurred in 30–55% of patients with HER2+ BC^1,2^, which suggests worse outcomes. CDK4/6 inhibitors have shown potential in overcoming resistance to HER2-targeted therapies by suppressing Rb phosphorylation and reducing TSC2 phosphorylation, thus resensitizing tumors to HER2 blockade^17^. Notably, p16^INK4A^, which can inhibit both CDK4 and CDK6^22^, has been found to be deficient in many HER2+ BC and BM patient-derived xenografts (PDXs). In these models, the combination of abemaciclib and tucatinib has led to significant tumor regression, suggesting that patients with HER2+ BC and BM could benefit from such combined treatments^23^. Prior to our study, DAP-HER-01 included 13 HER2+ BC patients (31.7%) with BM and 12 of them had untreated BM. The dalpiciclib and pyrotinib combination led to a CNS ORR of 66.7% (6/9) and a median PFS of 11.0 months^24^, aligning closely with our median PFS of 10.6 months, although our CNS ORR was lower at 35.7%. This discrepancy may partly arise from the fact that five patients with isolated BM progression in DAP-HER-01 underwent concurrent local stereotactic radiosurgery, which could influence the CNS ORR. Conversely, another study involving abemaciclib and trastuzumab did not yield confirmed intracranial responses, and the median intracranial PFS was a mere 2.7 months for HR+/HER2+ BC patients with BM^25^. However, this outcome is less compelling as only a small subset of patients (6/27) received the combination, with the majority on abemaciclib monotherapy. Given the small sample sizes and limited power in these subgroup analyses, the results should be approached with caution. Nevertheless, they offer tentative support for chemo-free regimens in managing HER2+ BC with BM. Considering the high risk of cerebral recurrence in HER2+ BC, our investigation into the effects of combining palbociclib, trastuzumab, and pyrotinib with fulvestrant on CNS disease control is both critical and timely. We now possess robust data regarding the efficacy and safety of HER2 TKIs for BM, enabling greater flexibility in designing future trials that incorporate combinations with antibody-drug conjugates (ADCs) or other therapeutic agents.

Remarkably, half of the patients (7 out of 14) were treatment naïve for metastatic disease, and all but two exhibited disease presence in both intracranial and extracranial sites. Yet, every patient who withdrew from the study experienced disease progression in both compartments, indicating that the quadruplet regimen demonstrates comparable activity within the brain and elsewhere in the body, with similar magnitude of benefit in the intra and extracranial compartments. This observation is further corroborated by the alignment of total ORR with CNS ORR and the proximity of PFS to CNS PFS. These findings make the study regimen interesting from the mechanism of action standpoint as a non-chemotherapy regimen.

The quadruple regimen demonstrated a favorable safety profile. Diarrhea was the most common AE of pyrotinib, while the incidence of grade ≥3 diarrhea in our study (39.7%) was consistent with pyrotinib combined with capecitabine (31% in PHOEBE^19^) and pyrotinib combined with trastuzumab and docetaxel (46% in PHILA^7^). This condition was effectively managed with dose adjustments and anti-diarrheal treatments, such as montmorillonite powder or loperamide, without leading to treatment discontinuation. Notably, the overall incidence of any grade ≥3 AEs in our study (46.7%) was considerably lower compared to the PHILA study (90%). Without chemotherapy, the percent of patients who experienced grade 3 or 4 AEs in pyrotinib, letrozole and dalpiciclib combination and dalpiciclib and pyrotinib combination were 68%^26^ and 95.1%^24^, respectively. No serious AEs were recorded in our study. These findings indicate that our chemo-free quadruple-combination regimen is well-tolerated and could serve as an alternative for patients seeking to avoid the adverse effects of traditional chemotherapy, such as alopecia, fatigue, and hepatic dysfunction, or for those who have suffered severe bone marrow suppression from multiple chemotherapy lines.

However, this study had its limitations. Firstly, it is a single-arm study without a control group and involves a relatively small patient cohort. More patients are currently recruiting. Secondly, drug concentrations in the cerebrospinal fluid were not measured, precluding an assessment of the study drugs’ penetration into the BBB. Thirdly, while the quadruple combination regimen in this study builds on previous research, a more robust approach might entail conducting preclinical or phase I studies to affirm the safety and efficacy of the combination from the outset. Fourthly, to maximize insights into the treatment’s impact on CNS lesions, the study currently excludes patients who have previously undergone WBRT or those whose intracranial target lesions were subject to local therapy due to the limited size of our cohort. Indeed, combining local with systemic therapies can extend control of the disease in those with a manageable CNS disease burden, potentially postponing the need for switching therapies amidst a stable extracranial response. Moving forward, we may consider including patients who have previously received WBRT or local treatment to broaden our study’s scope.

In conclusion, our preliminary results indicate that the combination of palbociclib, trastuzumab, and pyrotinib with fulvestrant is an effective treatment for patients with HR+/HER2+ MBC and BM, including those previously treated with anti-HER2 agents. The study is ongoing, and we anticipate providing updated results upon its completion. Given treatment challenges among patients with MBC with CNS involvement, our findings are particularly significant, demonstrating a promising CNS PFS with a chemo-free regimen. The publication of these interim findings contributes valuable insights into potential treatment strategies for HR+/HER2+ BC patients with BM.

## Methods

### Study design and patients

This was an investigator-initiated, multi-center, prospective trial conducted in three hospitals across China. A Simon two-stage design was used, with a one-sided alpha of 5% and power of 80%. The study was designed to enroll 34 patients across two phases. Among the 9 patients enrolled in the first phase, if 2 or more patients responded (complete response or partial response), the study would proceed to the second phase, enrolling the remaining 25 patients.

Eligible patients were those aged 18 years or older, with pathologically confirmed ER+ or PR+, HER2+ BC (ER/PR positivity defined as >1% tumor cell staining by immunohistochemistry; HER2 positivity defined as IHC 3+ or 2+ with a positive FISH test). Participants were required to have BM confirmed by MRI (both newly identified and previously existing lesions are qualified for enrollment), at least one measurable brain lesion (≥10 mm) as per the Response Evaluation Criteria In Solid Tumors 1.1 (RECIST 1.1), an Eastern Cooperative Oncology Group (ECOG) performance status of 0–2, a life expectancy of at least 12 weeks, no prior whole brain radiation therapy (WBRT), no stereotactic radiosurgery (SRS) or surgery of selected target lesion, and no systemic therapy other than trastuzumab within 2 weeks prior to initiating the study medication. Additionally, they must have demonstrated adequate organ and bone marrow function via blood tests. Exclusion criteria included meningeal metastasis, BM with severe complications (including challenging-to-manage neurological symptoms, brain herniation, brain edema or hemorrhage), and previous treatment with fulvestrant, everolimus, pyrotinib, or a CDK4/6 inhibitor. The complete list of eligibility criteria is detailed in the protocol.

The study received approval from the relevant institutional review board or ethics committee of Sun Yat-sen University Cancer Center, Guangxi Medical University Cancer Hospital, and National Cancer Hospital & Shenzhen Hospital and was conducted in accordance with good clinical practice guidelines and the Declaration of Helsinki. Written informed consent was obtained from all patients. This study is registered with ClinicalTrials.gov under the identifier NCT04334330.

### Procedures

In this study, patients were administered a regimen consisting of palbociclib, pyrotinib, trastuzumab, and fulvestrant. Specifically, patients received 125 mg of palbociclib orally once daily from day 1 to 21 of each 28-day cycle. This was combined with a continuous daily dose of 400 mg of oral pyrotinib. Trastuzumab was administered intravenously every 21 days, starting with an initial dose of 8 mg/kg, followed by a maintenance dose of 6 mg/kg. Fulvestrant was administered intramuscularly at a dose of 500 mg on day 1 and day 15 of each cycle. Patients will continue to be treated until tumor progression, unacceptable toxicity, or death occurs. To manage adverse events, dose reductions, and interruptions were permissible. Specifically, the palbociclib dose could be reduced in a stepwise manner from 125 mg to 100 mg and finally to 75 mg. Pyrotinib was allowed two dose reductions, first to 320 mg and then to 240 mg. No dose escalations were permitted following the resolution of toxicity. Delays in fulvestrant treatment of up to one week were allowed. The dose of trastuzumab could be adjusted only if the patient’s body weight varied by more than ±10% from the baseline. The protocol contains detailed guidelines for dose adjustments.

Radiological responses were evaluated every two treatment cycles, utilizing CT scans for extracranial lesions and MRI for CNS lesions. Both CNS and extra-CNS responses were assessed by investigators using RECIST 1.1 criteria. Routine blood tests, biochemical tests, and electrocardiograms were performed every two treatment cycles, while color Doppler echocardiography was conducted every three cycles. Treatment-emergent adverse events were monitored at each examination and graded according to the National Cancer Institute Common Terminology Criteria for Adverse Events (version 5.0).

### Outcomes

The primary endpoint was CNS ORR, defined as the proportion of patients with greater than or equal to 30% reduction in the CNS target lesions (calculated as the sum of the long diameters), without an increase in steroid use, clinical deterioration, emergence of new lesions, or progression of extracranial lesions according to RECIST 1.1 criteria.

Secondary endpoints included PFS (the time from randomization to the first radiographically confirmed disease progression or death due to any cause without documented progression), OS (the time from randomization to death), CNS ORR and non-CNS ORR (the proportion of patients showing complete response or partial response per RECIST 1.1), time to CNS progression, and safety of the treatment regimen.

Pyrotinib has been authorized for use in HER2+ MBC in China since October 12, 2018^20^. Consequently, a number of patients had already received pyrotinib as part of their treatment before this study, rendering them ineligible for enrollment, which led to a slower accrual rate.

### Statistical analysis

The results of this study will be presented through a detailed statistical description. Quantitative data, such as age, height, and weight, will be summarized using mean, standard deviation, median, maximum, and minimum values. Qualitative data, like gender and ECOG score, will be expressed in terms of frequency and percentage. For the primary endpoint, CNS ORR, and secondary endpoint ORR, we will calculate the incidence rate and provide a 95% confidence interval. Survival curves will be plotted using the Kaplan-Meier method for the secondary endpoints of PFS, OS, and time to CNS progression, and survival rate and 95% confidence intervals will be provided. All analyses were based on the safety analysis set, which included all patients who received at least one dose of study treatment. The safety analysis will be descriptive, mainly including the basic characteristics of subjects, adverse events, changes in vital signs, physical examination, and laboratory toxicities.

All statistical analyses will be calculated using SAS 9.2 statistical analysis software. Statistical tests will be two-sided, with a *P*-value threshold of ≤0.05 set for statistical significance and a 95% confidence interval applied.

### Supplementary information

Related Manuscript File

## Data Availability

All the data in this study are available from the corresponding author upon reasonable request. Qualified researchers may request access to patient-level data and related study documents, including the clinical study report, study protocol with any amendments, blank case report form, statistical analysis plan, and dataset specifications. Patient-level data will be anonymized, and study documents will be redacted to protect the privacy of our trial participants.
